# Idiopathic cerebrospinal fluid overproduction: case-based review of the pathophysiological mechanism implied in the cerebrospinal fluid production

**DOI:** 10.3325/cmj.2014.55.377

**Published:** 2014-08

**Authors:** Gianluca Trevisi, Paolo Frassanito, Concezio Di Rocco

**Affiliations:** Pediatric Neurosurgery, Catholic University Medical School, Rome, Italy

## Abstract

Cerebrospinal fluid (CSF) overproduction results from either CSF infection or choroid plexus hypertrophy or tumor, with only a single idiopathic case described so far. We report a unique case of a male infant with Crouzon syndrome who presented with intracranial hypertension, caused by up to 4-fold increase in CSF daily production. Conditions related to CSF overproduction, namely central nervous system infections and choroid plexus hypertrophy or tumor, were ruled out by repeated magnetic resonance imaging and CSF samples. Medical therapy failed to reduce CSF production and the patient underwent several shunting procedures, cranial expansion, and endoscopic coagulation of the choroid plexus. This article thoroughly reviews pertinent literature on CSF production mechanisms and possible therapeutic implications.

Hydrocephalus is generally defined as abnormal accumulation of cerebrospinal fluid (CSF) within the ventricles and subarachnoid spaces, and is often associated with dilatation of the ventricular system and increased intracranial pressure (ICP). It can be regarded as a disparity between production and absorption of cerebrospinal fluid (CSF), almost always as a result of CSF flow obstruction either at the ventricular level (eg, foramen of Monro, aqueduct of Sylvius, outlets of fourth ventricle) or the subarachnoid level (eg, basal cisterns, arachnoid granulations, or other points of absorption). More rarely hydrocephalus is a result of impaired absorption of CSF, due to increased venous pressure in the dural sinuses or at the jugular veins (1) and even more rarely of choroid plexus hyperplasia (2-4) or choroid plexus tumors (5-7). Furthermore, CSF production may be increased by CSF infections and meningitis (8), along with the concomitant decrease in CSF absorption. Hydrocephalus resulting from excessive production of CSF and not related to the conditions described above has been described in the literature by a single case report in 1989 (9).

We present a unique case of idiopathic CSF overproduction and the specific difficulties arising from its management. This case prompted us to review the literature focusing on the molecular mechanisms of CSF production and the drugs regulating this process.

## Case report

**Figure 1:**
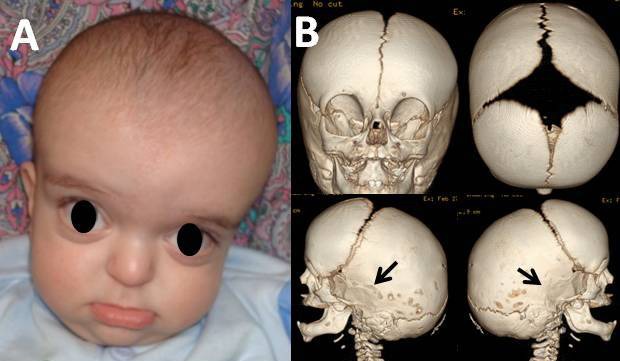
Patient picture at presentation (**A**). 3D reconstruction of computed tomography (CT) scan showed that the sutures of the cranial vault were widely open, while the squamosal suture, as well as parieto-mastoid suture, was fused bilaterally (arrows) (**B**).

A 6-month-old male infant, born to non-consanguineous parents, presented with macrocrania, irritability, and multiple episodes of vomiting. He also had dysmorphic features, in particular low-set ears, hypertelorism with exorbitism, mandibular prognatism with inverted bite, and broad and varus big toes. The clinical diagnosis of Crouzon syndrome was confirmed by a genetic test, showing FGFR2 gene mutation. Head computed tomography (CT) scan showed a premature fusion of the squamosal and parieto-mastoid suture bilaterally with open sutures at the cranial vault (Figure 1). Brain magnetic resonance imaging (MRI) showed a moderate ventriculomegaly with wide pericephalic subarachnoid spaces and a small-sized posterior cranial fossa with low insertion of the tentorium without Chiari malformation or crowded appearance of the neural structures. Angio-MR sequences showed a normal venous pattern with a slight predominance of the right transverse sinus. Prolonged monitoring (48 hours) of the ICP via an intraparenchymal sensor showed pathologically high values. Thus, a ventriculoperitoneal shunt (VPS) was placed, with prompt resolution of the preoperative symptoms. Intraoperative CSF samples showed no evidence of infections.

**Figure 2:**
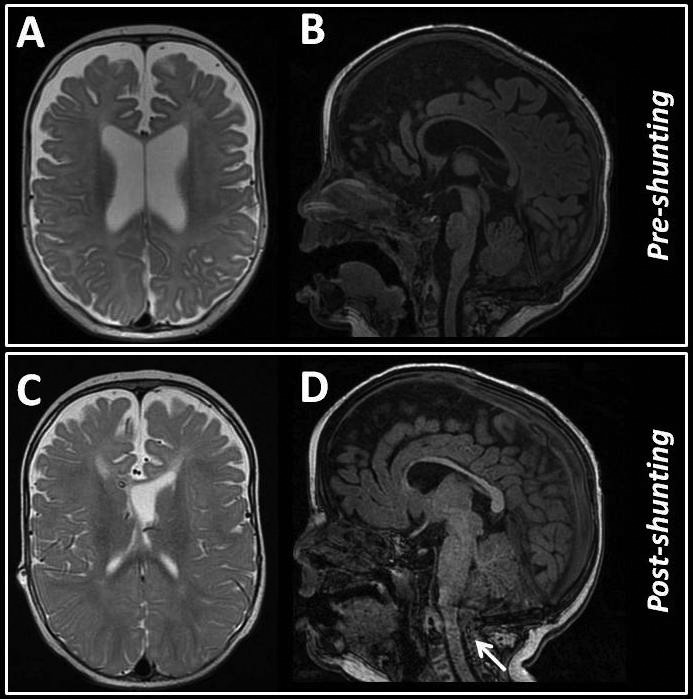
Brain magnetic resonance imaging (MRI) at presentation showed ventricular dilation without apparent obstruction of the cerebrospinal fluid circulation (**B**),(**C**). MRI performed two months later ruled out shunt malfunction, but disclosed a crowded posterior cranial fossa with Chiari I malformation (**C**),(**D**), arrow.

Two months later, the patient started vomiting daily. Pediatric work-up ruled out systemic issues. MRI did not show any VPS malfunction but showed craniocerebral disproportion, with crowded posterior cranial fossa and downward herniation of cerebellar tonsils **(**Figure 2). A posterior cranial expansion with decompression of the foramen magnum was performed. The postoperative period was complicated by CSF leak at the surgical wound, requiring exteriorization of the shunt. Unexpectedly, the average amount of drained CSF was 1200 mL/d. Patient did not present with any symptom and CT scan ruled out any complication of overdrainage. Repeated CSF samples ruled out any infection. No evidence of choroid plexus hypertrophy or tumor was present on MRI. The CSF production was not reduced by a trial of acetazolamide, but was increased to 1400 mL/d. Additionally, Colton blood group analysis showed a normal expression of Colton antigen, which is found on aquaporin 1 protein.

The subsequent placement of VPS was early complicated by massive ascites. Thus, we placed a ventriculoatrial shunt (VAS) and a concomitant “protective” external ventricular drainage (EVD). Transient closure of EVD, in order to challenge the cardio-vascular system to receive such a daily amount of CSF, was associated to an increased blood level of brain natriuretic peptide (BNP). Finally, two CSF shunt devices were concomitantly placed, namely a VPS on the left side and a VAS on the right side, both utilizing a middle pressure Pudenz valve.

**Figure 3:**
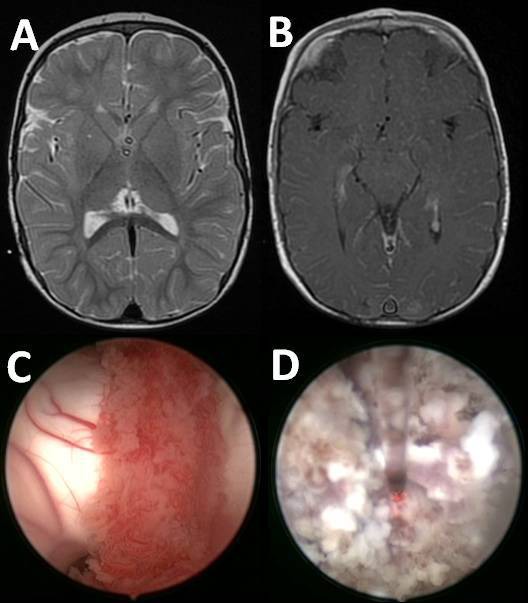
Brain magnetic resonance imaging (MRI) after Gadolinium administration ruled out pathological conditions affecting the choroid plexuses (**A**),(**B**). Endoscopic pictures of the choroid plexus before (**C**) and during (**D**) the coagulation performed using the Thulium laser.

A few months later, the child suffered again from symptoms of increased intracranial pressure. After surgical revision of both shunting devices, MRI confirmed craniocerebral disproportion with slit ventricles. A biparietal cranial expansion was therefore performed. However, postoperative CSF drainage still ranged between 1400 mL and 1700 mL per day, requiring once again dual shunting. After 5 weeks a new shunt malfunction required exteriorization of both CSF shunts, confirming daily overproduction of CSF (about 1500 mL/d). A pharmacological approach with terlipressin administration was attempted with poor results. Thus, in spite of normal appearance of the choroid plexuses at MRI, endoscopic choroid plexus coagulation was performed bilaterally. Due to small ventricular size, the procedure was performed through bilateral occipital access and using neuronavigation (Figure 3). Since the first post-operative day, the CSF drainage rate halved to 700-800 mL/d. In spite of this important reduction of the CSF production rate, it was necessary to perform dual shunting. The postoperative course was uneventful and the patient was doing well at 6-month follow-up.

## Discussion

Hydrocephalus resulting from CSF overproduction is a rare disease and is usually caused by pathological conditions affecting the choroid plexus, namely hyperplasia or tumor. In the last decade, endoscopic coagulation of the choroid plexus has become a treatment option for this condition, warranting shunt independency, with lower complication rate compared to surgical plexectomy (10). In the case of choroid plexus tumor, the surgical resection of the tumor usually reduces CSF production. Persistent hydrocephalus, requiring the placement of a CSF shunting device, is possibly related to the concomitant impaired absorption of CSF, ascribed to chemical arachnoiditis secondary to the presence of the tumor in the ventricular system (11,12). Similarly, CSF infections and meningitis (8) may increase the CSF production, along with the concomitant decrease in CSF absorption. Antibiotic treatment of the infection usually leads to normalization of CSF production but not necessarily to the treatment of the associated hydrocephalus, since the structures devoted to the CSF absorption may be definitely impaired, thus usually requiring a shunting procedure.

To our knowledge, a single case of idiopathic CSF overproduction, ie, not related to the conditions described above, has been reported in the English-language literature so far (9). In this case from 1989, increased daily production of CSF could have been exacerbated by the infections that the patient had suffered from in the months before the reported CSF output records. Thus, hydrocephalus related to idiopathic CSF overproduction remains a questionable pathological entity.

In our case, there was no evidence of CSF infection in the patient’s clinical history. The patient with Crouzon syndrome suffered from a communicating hydrocephalus secondary to impressive idiopathic CSF overproduction. In patients with Crouzon syndrome, hydrocephalus is present in up to 40% of cases and is usually related to venous outlet occlusion and small posterior cranial fossa (13). In the present case, the initial MRI showed normal dural sinuses anatomy, and the posterior cranial fossa was not particularly small and crowded. Serial MRI performed to investigate the cause of CSF overproduction ruled out concomitant pathological conditions affecting the choroid plexuses. Interestingly, the patient did well over two months after the initial placement of a CSF shunt, so that it is possible that the device placement precipitated CSF overproduction via an unknown mechanism. The absence of underlying conditions causing the CSF overproduction together with the young age of the patient and his comorbidities raised particular issues in the management of this case. The placement of VPS was complicated by massive ascites, as expected. Although in the previous case report, the 2.5-year-old child with more than four times the expected rate of CSF production was successfully treated with a VAS (9), in the present case the cardiovascular system did not tolerate such a daily amount of CSF, so that we finally placed a VAS on the right side and a VPS on the left side. Cardiac function was thereafter monitored by ultrasound and weekly blood level of BNP in order to promptly rule out any volume overload. Unfortunately, the presence of two CSF shunt devices reinforced the spontaneous tendency of the cranial vault sutures to fuse due to the underlying syndrome condition, thereby leading to the development of secondary craniosynostosis and craniocerebral disproportion (14). In spite of surgical expansion of the cranial vault, the ventricles remained particularly small, thus resulting in a constant risk of shunt obstruction.

Finally, the endoscopic coagulation of the choroid plexus, though initially contraindicated due to the risk related to the small size of the ventricular cavities, was performed as a last resort option when the other treatment options failed. This procedure resulted in a significant reduction of CSF production, thus confirming its role in complicated cases of hydrocephalus (15).

This unique case of idiopathic CSF overproduction prompted us to review the mechanisms of CSF production, which are still far from being completely understood, in order to identify possible targets of a medical therapy.

### CSF production

CSF production is an active process, which according to the classical theory takes place mainly in the epithelial cells of the choroid plexuses (16-19). The driving force for fluid secretion is an osmotic gradient due to the active and unidirectional flux of ions from one side of the epithelial monolayer to the other (20). The main ions and small molecules secreted by the choroid plexus through transporters and ion channels are Na^+^, Cl^-^, HCO_3_^-^ and H_2_0, while K^+^ ions are absorbed (21,22) **(**Table 1**)**.

Moreover, there is active water secretion through transmembrane proteins referred to as aquaporins (AQPs) (39). Among the 13 known mammalian AQPs, five have been identified in the central nervous system (CNS), namely AQP1, AQP4, AQP7, AQP9, and AQP11 (40).

Although the mechanism of ion secretion has been clarified and most of the involved transporters have now been localized to specific membranes (21,22), the role of AQPs in CSF physiology and pathophysiology is still uncertain. AQP 1 and 4 are the most expressed subtypes in the mammalian CNS. AQP1 is highly expressed at the apical surface of the choroid plexus epithelium (39,41) whereas AQP4, the most abundant water channel in the brain, is expressed by ependymal cells, glia limitans interna and externa cells, at the perivascular end feet of astrocytes that form the blood-brain barrier, and in the cerebellum, supraoptic and suprachiasmatic nucleus of the hypothalamus, hippocampal dentate gyrus and areas CA-1 and CA-3, neocortex, nucleus of stria terminalis, and medial habenular nucleus (40).

Distribution of AQP1 at the choroid plexus apical surface suggests its role in CSF production. Studies on AQP1 knockout mice showed a decrease in both CSF production and ICP values compared with wild-type mice, although the latter effect could be related to the decrease in the central venous pressure in AQP1 knockouts due to the effect on the kidneys (42,43). A recent literature search for articles on the role of AQP1 in hydrocephalus found just 5 papers, 3 on animal models and 2 on humans (44). Down-regulation of AQP1 has been reported in animal studies and in a case report on a 15-month-old girl with choroid plexus hyperplasia (45), while a small case series of choroid plexus tumors showed a heterogeneous pattern of AQP1 in papillomas and a weak expression in a single case of choroid plexus carcinoma (46). These data suggest a possible existence of a feedback mechanism on AQP1 expression in response to hydrocephalus (40). In our case, chemical analysis of the CSF showed a normal glucose level, very low protein values (3-5 mg/dL), and absent cells in the patient’s clinical history. These data are consistent with active secretion of water at the ventricular level in spite of very low oncotic pressure of the CSF. Thus, even if no clinical case of hydrocephalus has been related to a pure deficiency of aquaporins, we performed a molecular analysis of Colton antigen expression during blood tests (Colton antigen is found on AQP1), in order to investigate any mutation of AQP1. However, this test did not show significant anomalies.

Studies on AQP4 role in brain edema in knockout mice models showed a protective role of the protein in vasogenic edema development but an active role in cytotoxic edema development (47,48). Animal hydrocephalus models showed an up-regulation of AQP4 expression in the blood-CSF and blood-brain barriers, suggesting the presence of an adaptive feedback mechanism that lowers the production and increases the clearance of CSF in cases when trans-parenchymal flow and hydrocephalic edema are observed (40). Recently, an increase in AQP4 has been reported in the CSF in human congenital hydrocephalus, which could be the result of ependyma denudation and subsequent diffusion of AQP4 from the parenchyma to the CSF (49). Recent data has focused on the role of AQP4, which is mainly distributed in the perivascular spaces, in the regulation of CSF homeostasis (50). Moreover, AQP4 deficit has been shown to cause hydrocephalus in experimental models (50).

In conclusion, the traditional explanation that the CSF is produced by the choroid plexuses and reabsorbed by the arachnoid villi has been questioned. A study in children hypothesized the presence of a minor pathway involving the perivascular spaces as a site of CSF reabsorption (51). The present case of persistent overproduction of CSF after the coagulation of the choroid plexuses further brings into question the origin of the CSF. Recent insights have shifted the attention to the microvessels and their perivascular spaces, as sites responsible of the establishment of homeostasis between CSF and the interstitial fluid, questioning the net CSF production by the choroid plexus (52,53).

### Regulation of CSF production

Different molecules have been implicated in the regulation of CSF production **(**Table 2**)**. One of the corticosteroid action mechanisms for reducing brain edema and ICP is the reduction of CSF production mediated by a decreased activity of Na^+^-K^+^ATPase (54).

Similarly, CSF production may be influenced by anesthetic agents (55). Halothane decreases CSF production by acting on the V1 receptor of vasopressin, with no net change in the blood flow to the choroid plexus (56). Other agents, such as thiopental, midazolam, and etomidate, may decrease CSF production by reducing both cerebral blood flow (CBF) and cerebral metabolic rate for oxygen when used in high doses (57). Although opioid anesthetics, including fentanyl, can increase ICP (58-60) and are generally believed not to influence CSF production (61), high doses of fentanyl have been reported to reduce CSF production in dogs (62). As opposed to this, ketamine increases the CSF production secondary to an increase in CBF (63). Enflurane, despite a reduced CBF, causes an increase in choroid plexus metabolism, resulting in an increased CSF production (64). Moreover it increases the resistance to reabsorption of CSF, which together with increased CSF production, contributes to increasing ICP (56,65).

Interestingly, a widely used substance such as caffeine, in rats showed opposite effects on CSF production: acute caffeine treatment (oral intake) reduced CSF production, while a chronic intake resulted in ventriculomegaly, due to increased CSF production and potentially associated periventricular leukomalacia (66). Such an effect of caffeine is mediated by A_1_ adenosine receptor. The increased CSF production is the result of an increased activity of Na^+^-K^+^ ATPase at the choroid plexus level, which establishes a sodium gradient across the choroid epithelial cells, as well as of an elevated CBF (66).

These molecules are not used in neurosurgical practice to regulate the CSF production, but acetazolamide, a carbonic anhydrase inhibitor, has been used for a long time to reduce CSF production in idiopathic intracranial hypertension. In vivo studies after administration of acetazolamide showed a 50% decrease in CSF production despite a 2-fold increase in blood flow to choroid plexus (67). Carbonic anhydrases are a group of enzymes that catalyze the production of HCO_3_^-^ and H^+^ from H_2_O and CO_2_. Although carbonic anhydrases are not directly involved in ion transport, they are believed to have an important role in CSF secretion on the basis of the effects of their inhibitors (21). Unfortunately, acetazolamide is not always effective and its long-term use can be difficult to tolerate by both adults and children. In children, potential side effects are lethargy, poor feeding, tachypnea, diarrhea, and electrolyte imbalances (hyperchloremic metabolic acidosis). Finally, a paradoxical increase in CSF production may be observed after acetazolamide administration because of increased levels of circulating CO_2_ and subsequent vasodilatation and increased cerebral blood flow, as observed in the present case. Another drug that has a weak carbonic anhydrase inhibition activity is the widely used loop diuretic furosemide, which has also been shown to decrease CSF production (68). Similarly, bumetanide, another loop diuretic, blocks Na^+^-K^+^ -2Cl^-^ cotransporter, thus showing a significant reduction (47%) in CSF production in animal models (69). Interestingly, butamenide also showed an inhibitory effect on AQP1 and AQP4 (70). This finding led to the development of molecules based on bumetanide structure like arylsulphonamide AqB013 as an antagonist for AQP1 and AQP4 (71). It is worth to note that acetazolamide is also part of arylsulphonamide family. Although its potential effects on AQPs are still debated (72), recent studies have shown an interaction with AQP1, both in vitro (73) and in vivo (74), and a possible reversible inhibition of AQP4 (75).

Experimental data showed that circulating antidiuretic hormone (ADH), also known as vasopressin, markedly decreased CSF production in rabbits (76) and sheep (77). This effect is presumably related to choroid plexus arterioles vasoconstriction mediated by vasopressin V_1a_ receptor, which has been localized on the choroidal tissue by both autoradiography and mRNA analysis (78,79). On the contrary, vasopressin V_1b_ receptors, which mainly mediate corticotrophin secretion from the pituitary, are less expressed in choroid plexus tissue. However, an increment in V_1b_R mRNA was documented after salt-loading, suggesting a role in the regulation of brain water content and cerebral edema (80).

The role of endogenous vasopressin in CSF is still debated and is probably related to brain water regulation (80). It may arise from three sources: transport into the CSF from peripheral blood (81,82), central release from hypothalamic neurons (83), and in situ synthesis and release into the CSF from epithelial cells of the choroid plexus (84-86). Vasopressin production by the choroid plexus is increased by beta-adrenergic agonists and vasointestinal peptide, which indicates that the process is under both endocrine and neuronal control (87). Therefore, vasopressin could have an autocrine and paracrine effect, playing a role in the chain of events that enables either hormonal or neuronal influence on the control of CSF production (20). In the present case report, an off-label medical trial with low dosage intravenous terlipressin was started with the parents’ consent. Terlipressin is an analogue of vasopressin, acting on three different receptors: vasopressin receptor V1a, vasopressin receptor V1b, and, less actively, on vasopressin receptor V2, which controls free water absorption in the renal medulla. Its current clinical use is in septic shock (88), hepatorenal syndrome (89), and esophageal varices bleeding (90). In the present case, this medical therapy had a promising effect of halving the daily amount of drained CSF since the third day of therapy and the child maintained a good clinical status without hemodynamic complications. However, due to the risks related to a prolonged vasoconstrictor therapy, terlipressin was stopped after 6 days with a new increase in the drained CSF volume to pre-therapy levels without chemical or microbiological alterations. Since the administration modality and potential risks of cardiovascular disease and fluid imbalance do not make terlipressin an ideal candidate for long-term therapy, further studies should identify most manageable and selective analogues of vasopressin.

## Conclusions

CSF production is a complex and active process that involves a huge number of transporters, with insufficiently understood regulatory mechanism, which makes the management of idiopathic CSF overproduction a complicated issue. This extremely rare pathological condition could be related to the malfunction of ions or water transporters at ependymal and choroid plexus level or to an imbalance in the CSF production regulatory mechanism. Drug therapy has so far failed to control the CSF production, so that surgical and endoscopic options, in particular choroid plexus coagulation, should be considered. A better understanding of the molecular substrates of the CSF production could lead to the development of targeted drugs that could reduce CSF production and alleviate intracranial hypertension symptoms in hydrocephalic patients and potentially have a role in reducing brain edema related to ischemic and neoplastic lesions.

## 

**Table 1:** Main ion transporters and channels expressed in mammalian choroid plexus (CP)

Transporter	Route of action	Site of expression in CP cells	References
Na^+^-K^+^ATPase	Na^+^ secretion K^+^ absorption	Apical membrane (3 isoforms)	Zlokovic et al 1993 (23)
Na^+^-K^+^ -2Cl^-^ cotransporter	Still debated, probably absorption of Na^+^, Cl^-^ and K^+^	Apical membrane	Wu et al 1998 (24), Angelow et al 2003 (25)
K^+^-Cl^-^ cotransporters	K^+^ and Cl^-^ secretion	KCC3 isoform: Basolateral membrane KCC4 isoform: Apical membrane	Pearson et al 2001 (26)
Cl^-^-HCO3^-^ exchange	HCO3^-^ secretion Cl^-^ absorption	Basolateral membrane	Johanson et al 1990 (27)
Na^+^- HCO3^-^ cotransporters	Isoforms NBCn1 and NBCE: Na^+^ and HCO3^-^ absorption Isoform NBC4g: HCO3^-^ secretion	NBCn1 and NBCE: Basolateral membrane NBC4g: Apical membrane	Praetorius et al 2004 (28); Fukuda et al 2013 (29)
AQP1	Probably H_2_O secretion Cation channel: Cs^+^, Na^+^, K^+^, and, to a lesser extent, TEA^+^ absorption	Apical membrane	Nielsen et al 1993 (30); Boassa et al 2006 (31)
AQP4	To be determined	Cytoplasmatic organelles membranes	Venero et al 1999 (32)
Kv channels	K^+^ secretion	Apical membrane (3 isoforms)	Speake et al 2004 (33)
Kir channels	K^+^ secretion	Apical membrane	Döring et al 1998 (34); Nakamura et al 1999 (35)
Anion channels	Inward-rectifying channels and volume-conductance channels: probably HCO3^-^ and Cl^-^ secretion	Not known (theoretical site: Apical membrane)	Speake et al 2000 (36)
TRP channels	TRPV4 and TRPM3: non-selective cation channels that increase transcellular ion flux and paracellular permeability to adjust to changes in extracellular osmolarity	Not known	Liedtke et al 2000 (37); Jo et al 2013 (38)

**Table 2:** Drugs that may influence CSF production at choroid plexus level and their proposed mechanism of action

Drug	Effect on CSF production	Proposed mechanism of action
Corticosteroids	Reduced	Reduced Na^+^-K^+^ATPase activity
Acetazolamide	Reduced	Carbonic anhydrase inhibitor Interactions with AQP1 and AQP4
Furosemide	Reduced	Weak carbonic anhydrase inhibitor
Bumetanide	Reduced	Blocks Na^+^-K^+^ -2Cl^-^ cotransporter Inhibitory effect on AQP1 and AQP4
Vasopressin	Reduced	Choroid plexus arterioles vasoconstriction
Halotane	Reduced	Interaction with vasopressin V1 receptor
Thiopental (high doses)	Reduced	Reduction of CBF and cerebral metabolic rate for oxygen
Midazolam (high doses)	Reduced	Reduction of CBF and cerebral metabolic rate for oxygen
Etomidate (high doses)	Reduced	Reduction of CBF and cerebral metabolic rate for oxygen
Fentanyl (high doses)	Reduced	Not determined
Caffeine	Acute effect: Reduced Chronic effect: Increased	A_1_ adenosine receptor mediated increased Na^+^-K^+^ATPase activity + Increased CBF
Ketamine	Increased	Increase in cerebral blood flow
Enflurane	Increased	Increase in choroid plexus metabolism

*Abbreviations: AQP – aquaporin; CBF – cerebral blood flow.
